# Notes and Comments

**Published:** 1894-05

**Authors:** 


### Notes and Comments.[1]

THE CAUSE OF HYPNOSIS.—We have had numerous essays and discussions in the societies, the journals, and the press upon the subject of hypnotism, but the point of most interest and concern to many of us has not been reached,—that is, a satisfactory explanation of these phenomena. In this respect very little advance has

---

[1] The assistant editor solicits contributions for this department,—new methods, new remedies and formulas, or any short practical note which may prove of value to the practitioner or student. Address 212 South Fifteenth Street, Philadelphia.

been made for the last quarter of a century or more. When we come to seek the source, the cause commensurate to the effect, it has evidently baffled the investigator and seemed as inexplicable as the fundamental problem of human existence, "What is life?"

At a recent meeting of the Odontological Society of Pennsylvania the psychic side of hypnosis was touched upon by several, and when the evidence is all in I am confident the verdict will be, that this recognized force in man (call it the unknowable) has much to do with all these manifestations.

It was only a few years ago that many of our scientific men, especially our physiologists, seemed moving towards a material explanation of all things. They tell us that the phenomena of perception is caused by a movement in the cells of the optic thalami, while that of the memory is caused by a movement in the cells of the cortical portion of the cerebrum. These cells, which are intimately associated, respond to special excitement and give rise to the phenomena known as recollection and association of ideas.

This can be demonstrated; but can such physiologists explain the force back of cell-life? Can the chemist's retort or the biologist's culture-tube solve the secrets of animal creation? No; but, on the other hand, we could marshal an imposing array of witnesses who express the conviction that "we are now nearly everywhere compelled to assume a specific yet unknown activity of the living cell." With this frame of mind—as a recent issue of the Philadelphia *Press* has it—comes an increasing conviction that no material explanation of the universe is adequate or logical, a growing belief that a power must be accepted as enwrapping, supporting, and rendering vital the universe of matter and the world of mind.

